# Maternal and child health indicators in primary healthcare facilities: Findings in a health systems quasi-experimental study in western Kenya

**DOI:** 10.1016/j.dialog.2023.100133

**Published:** 2023-04-10

**Authors:** Fabian Esamai, Ann Mwangi, Mabel Nangami, John Tabu, David Ayuku, Edwin Were

**Affiliations:** a Dept of Child Health and Paediatrics, School of Medicine College of Health Sciences Moi University, Kenya; b Dept of Behavioural Sciences, School of Medicine College of Health Sciences Moi University, P. O Box 4606, 30100 Eldoret, Kenya; c Dept of Health management and Health Policy, School of Public Health, College of Health Sciences, Moi University, P. O Box 4606, 30100 Eldoret, Kenya; d Dept of Disaster Risk Management, School of Public health, College of Health Sciences, Moi University, P. O Box 4606, 30100 Eldoret, Kenya; e Dept of Reproductive Health, School of Medicine College of Health Sciences Moi University, P. O Box 4606, 30100 Eldoret, Kenya

**Keywords:** Health systems, Maternal, Neonatal, Enhanced Health Care, Find link treat and retain

## Abstract

**Background and purpose:**

Maternal and infant mortality are higher in low-income than in high-income countries due to weak health systems. The objective of this study was to improve access, utilization and quality of Maternal and Child Health care through a predesigned Enhanced Health Care System (EHC) that embodies the World Health Organization (WHO) pillars of the health system.

**Design and methodology:**

This study was conducted in two dispensaries in the Counties of Busia and Bungoma in Kenya as intervention sites and in four control clusters in Kakamega, Uasin Gishu, Trans Nzoia and Elgeyo Marakwet Counties. The study population was pregnant women and their children delivered over the study period in the intervention and control clusters.

A quasi-experimental study design was used to conduct the study between 2015 and 2020 to compare the outcomes of the implementation of the EHC using the Find Link Treat and Retain (FLTR) strategy in one cluster, community owned initiatives in the other cluster and four control clusters at baseline and at the end of the study. A baseline survey was conducted in year one and an end line survey in the fifth year. Continuous data collection on maternal and childhood health indicators was done in all the six clusters and comparison made at the end of the study between the clusters.

**Results:**

We found a 26%, 10.3% and 0.8% increase in antenatal care (ANC) attendance in the intervention clusters of Obekai, Kabula and control clusters respectively. There was a 28.2%, 5.8% and 17.0% increase in attendance of 4+ ANC clinics of Obekai, Kabula and control clusters respectively. There was a 24% and 13% increase in Obekai and Kabula respectively in contraceptive use and a 2% decrease in contraceptive use in the control locations. There was a 38.2%, 25.6% and 34.7% increase in facility deliveries over the study period in Obekai, Kabula and control clusters respectively. There was a marked increase in immunization coverage in the intervention clusters of Obekai and Kabula compared to a significant decrease in control clusters for BCG, polio, pentavalent and measles.

**Conclusions and recommendations:**

In conclusion, use of the health systems approach in health care provision provides a holistic improvement in access and utilization of health services and in the improvement of health indicators.

We do recommend that a systems approach be used in health services delivery to improve access, utilization and quality of health care provision at community and primary care levels.

## 1 Background

Maternal and neonatal mortality are still unacceptably high in Sub-Saharan Africa and countries of South East Asia compared to Western Europe and Northern America (AMANHI mortality research group, 2018). In low-income settings, maternal mortality rates range from 150 to more than 1000 per 100,000 live births while rates of stillbirth and neonatal mortality generally range from 20 to 40 per 1000 births [1,2]. Intrapartum stillbirth or those stillbirths that occur during labor and delivery are an important indicator of the quality of obstetric care [3,4]. While in high-income countries intrapartum stillbirths have nearly been eliminated, in low-resource settings up to half of all stillbirths occur in the intrapartum period. UNICEF estimates neonatal mortality in developed countries to be as low as 5/1000 live births while in developing countries it is as high as 35/1000 live births [5,19].

One emerging challenge to achieving and sustaining desired targets for maternal and child health especially in low and middle income countries is the neglect in addressing the gap between the community and primary care health facilities. In addition there is failure to espouse a health systems approach as well as the continuum of the Three Delays Model [6] in health care provision in these countries.

To achieve the concept of Universal Health Coverage as recommended by the World Health Organization and the World Health Assembly, countries need to embrace the implementation of health services that include the pillars of health systems of the WHO [[7], [8], [9]]. These pillars include service delivery, human resources, health management information systems, medical products and supplies, financing and leadership and governance [8,10].

Our hypothesis in this study was that the implementation of enhanced health care (EHC) through the Find Link Treat and Retain (FLTR) strategy, the implementation of the WHO pillars and the three delays Model in the intervention clusters, shall lead to a significant increase in antenatal care attendance, increase in health facility delivery in the intervention clusters than in control clusters. We also hypothesize this will lead to improved access and utilization of health care services in the intervention clusters and facilities (increased general attendance in the facility and increased immunization coverage) than in control clusters and facilities. In addition we expected a greater reduction in maternal and neonatal mortality in the intervention clusters than in control clusters. Furthermore, we did expect improved quality of care in the intervention clusters than in the control clusters.

The intervention to be tested is the implementation process of an enhanced health care (EHC) delivery package. *The EHC package comprises of the following components which address access, utilization and improved quality of health care at community level and in the facilities:*

• Early identification and surveillance of the pregnant woman and newborn babies and referral to the facility.
• Recognition and management of pregnancy-related complications, particularly pre-eclampsia, hemorrhage, premature rupture of membranes and infection.
• Recognition and treatment of underlying or concurrent illness in pregnancy – malaria, anemia, infection among others.
• Screening for conditions and diseases such as syphilis, HIV infection and hepatitis B.
• Preventive measures through de-worming, administration of iron and folic acid supplements, intermittent preventive treatment of malaria in pregnancy (IPT), prenatal vitamins provision and use of Long Lasting Insecticides Treated Nets (LLITNs).
• Developing a birth and emergency preparedness plan.
• Immunization of all young infants and under-five children born during the study period including Vitamin A administration at 6 months of age and yearly.
• Obtain weights for all newborns, infants, under five year old children and pregnant mothers in the community and health facilities by providing weighing scales (infant, adult).
• Malaria diagnosis using the rapid diagnostic tests (RDTs) in the community and health facility.
• Hemoglobin estimation using Haemocue in the community and at the facility.

## 2 Project goal

Improve maternal and child healthcare in Kenya through health systems strengthening initiatives at community and primary health care levels.

## 3 Specific objectives

There were six specific objectives with objective 1 and 6 covering baseline and end line data collection. This enabled us to collect baseline data with which to compare the arms at the end of the study after 5 years [10].

The second objective was the main implementation arm of the study which was implemented in one cluster through project funds and supervision. This is the objective through which the hypothesis of this study is measured. This was conducted in Obekai dispensary [11].

The third objective was a community owned and driven objective in which the Kabula community implemented the program at their own cost and efforts [12].

The fourth objective was the implementation of a community strategy (FLTR) of Finding women, **L**inking them to the facility, **T**reating them or providing services at that facility and **R**etaining them in the facility health service through a planned follow up agreed with the women. This strategy was used for objective 2 in Obekai by the study team and in objective 3 in Kabula by the community with assistance from the Kabula dispensary health workers ([10,11], and [12]).

The fifth objective was a training component aimed at training health systems specialists in Kenya.

The following parameters were evaluated at baseline and at end line in the 1^st^and 5^th^ year of the study which constituted objectives 1 and 6:

• Increase in antenatal care attendance by pregnant women in the health facilities
• Increase in proportion and number of women delivering in health facilities
• Reduction in the rate of low birth weight and premature babies born
• Increase in overall immunization coverage and individual vaccine coverage
• Reduction in the rates of poor pregnancy outcomes including birth asphyxia, neonatal sepsis, neonatal mortality, maternal mortality, near miss maternal morbidity among pregnant women
• Exclusive breastfeeding rate, Infant and Young Children Feeding (IYCF) and IMCI parameters, anthropometric indicators.

Findings from this study for Objectives 2, 3 and 4 have been published [11,12] in which we found trends in improvement of facility utilization and access in Obekai and Kabula at the end of the study period compared to baseline at the start of the study. We also found improvement in the quality of health care in Obekai dispensary after the implementation of the health systems approach compared to findings at the start of the study.

This paper addresses the differences observed in the three arms of the study with regard to access, utilization and improved health care as described in the protocol. We also analyze, compare and discuss some of the maternal and children’s health indicators for the three arms, in the results and discussion sections of this manuscript. We have used only the quantitative data in this analysis.

Objective five addressed lack of trained health workers in health systems and we aimed at building capacity in the field of health systems in which six master’s graduates were trained and one doctorate graduated over the 5 year study period.

## 4 Methods

We implemented this quasi-experimental community trial between January 2015 and September 2020 in 6 locations in 6 Counties in Kenya with 2 intervention sites and 4 controls. This design allowed a comparison of outcomes between the intervention and control arms. The details on how the study was implemented and findings in the objectives 2, 3 and 4 have been published [[10], [11], [12]].

***Baseline Assessment*** was conducted as per protocol which has been published [10]. The aim was to map out the structure and functions of the primary health care system at levels 1 and 2 of the Kenya health system. Maternal and infant outcomes were assessed including ANC attendance indicators, Facility delivery, and Signal functions for BeMONC, existence of nutritional, immunization and other IMCI services for under-five year old children.

**Baseline and end line data** collection forms were developed and data collected and entered into a data base in the computer and analysed using the STATA version 12.

To assess maternal and child health indicators, the status of the health system and existing programs in the study areas, descriptive statistics were used for the quantitative data and these are outlined in the results section of this paper.

**The *Intervention*** was designed and implemented as the EHC package using the FLTR strategy to improve access, utilisation and quality of care of maternal and child health at tier 1 and 2 in one intervention cluster.

For the implementation of the intervention model for objective 2 we utilised the FLTR (Find, Link, Treat, and Retain) strategy to support a successful implementation of Enhanced Health Care (EHC). This required direct investment in health system elements that contribute to efficiency of the referral system between the community and primary care levels and the County referral facilities under the new 4-tier health system in Kenya.

The FLTR (**F**ind **L**ink **T**reat **R**etain) strategy was used in the study to enable continuous care and follow up of the women and infants.

The **Find** in the FLTR strategy Involved investment in the Human Resources for Health (HRH) component of the health system by influencing recruitment of adequate and competent CHWs from within intervention villages and skilled health workers (SHWs) to follow up the women and infants. It covered investment in improvement in management components through strengthening of supervision by trained health workers through the use of appropriate technologies and supplies (computers, HMIS, mobile phone and household registers) to improve efficiency and strengthen the health information system.

The **Link** in the FLTR strategy used CHWs to engage and encourage prompt ANC/MCH clinic attendance and schedule ANC/MCH clinic appointments using mobile phone devices at the time of initial CHW visit (Link). This links directly to efficiency in Service delivery, investment in technologies (mobile phone) and health information for efficient management of referrals. This created a link between level 1 and level 2 of the health system. This therefore linked the community to the level 2 health facility (Obekai dispensary).

The **Treat** in the FLTR strategy was the service provision to the participants through ANC/MCH clinics in the facilities that provided the complete EHC package to each subject and addressed the quality of service delivery, technologies and commodities component. These included hemoglobin testing using the haemocue machine and malaria test using the Rapid Diagnostics Tests (RDTs). Any ailments were treated at the local facility and where referral was needed they were referred to the higher level facility. Newborns were given the routine vaccinations, weights taken and prenatal and vitamin A provided including all the relevant services as outlined in the EHC package.

We invested in improvement of quality of care through refresher training of all the skilled health workers in the study dispensary on MNCH, BeMONC, Essential Newborn care (ENC), customer care including public relations using the Ministry of health guidelines. The community health workers (CHWs) were also trained using the WHO guidelines on pregnancy surveillance and newborn follow up. This included methods of approach to household and villages and confidentiality. We provided essential equipment, drugs and dressings as per the essential commodities list of the Ministry of Health for the level 2 facility. This ensured that there were no stock outs and the related health systems pillar was effectively covered.

We utilized the modified Ministry of Health (MOH) Standard Operating Procedures for this level of care for the management of illnesses and provided acceptable waiting time for patients as recommended. In addition we conducted exit surveys for all mothers who were treated at this facility as they left the facility on the same day.

The **Retain** in the FLTR strategy ensured that subjects were tracked and those who default were traced by the CHW at the subject’s home. They were counseled and brought to the facility for the ANC/MCH visit. This component of the intervention links to building efficient and effective health information system that allows monitoring/tracking of subjects at antenatal, delivery and postnatal care. The component also ensures cost-reduction in tracking by applying appropriate technologies such as use of mobile phones. The follow up therefore ranged from 6 months to 3 years depending on the month and year of birth over the 5 year study period. This translates into the retention of the women and children in the health system throughout the period of the study.

This FLTR strategy was mainly applied to enroll pregnant mothers and their infants within the intervention cluster (Obekai dispensary). Women who became pregnant for the second or third time during the study period were enrolled and followed up as per protocol.

**Implementation of the *Innovative partnerships for objective 3 was by*** exploration and facilitation in partnerships with the community for innovative approaches (IGUs) to incentivize CHWs and Community Midwives (CMWs) to effectively participate in increasing access and retention of pregnant women and children in the health care system in the intervention clusters.

In this one cluster, Kabula in Bungoma, we worked with the community Health Units, village elders, Assistant chiefs and the Chief in collaboration with the community health extension workers (CHEWs) and community health workers (CHWs) to establish income generating units (IGUs) and/or community based organizations (CBOs) in the cluster. The IGUs/CBO were set up by the communities themselves and run through the governance structures established by the communities themselves. The funds from these IGUs/CBO were used to incentivize the volunteer CHWs to identify pregnant women and their newborns to the local dispensary. The Kabula primary care health facility committee worked with these CBO and IGUs to assist in the funding of some activities including referral costs from the community to the primary care health facility and to the county referral facilities. These IGUs/CBOs were used to address the transport and birth plans requirements of the pregnant women, their newborns and infants born during the life of the project.

**The Implementation of *referral system in objective 4 was to*** assess the effectiveness and sustainability of the implementation of FLTR and EHC in the improvement of the efficiency of the referral system between the community (level 1), primary care facilities (level 2) and County referral facilities (level 3) for pregnant women in the intervention area. The effectiveness and efficiency of the referral systems was evaluated in the intervention clusters and compared at baseline to end line in the two clusters where interventions were applied.

The number of referrals, timeliness of referrals, outcomes of referrals in terms of deaths and feedback provided to the referring facility by the referral facility, access of the referred patients to the doctors and consultants, transport availability and access, availability of referral notes and the detail of the referral and the feedback to the referring facility were evaluated. Interaction between the referring and referral facility in terms of visits, calls, letters, e mails and frequency of supervisory visits were recorded.

### 4.1 Study population

The study population was all pregnant women living within the catchment population of the dispensary (Cluster), their new-born babies and the under-five year old children born during the life of the project.

Two clusters were randomly selected as the intervention clusters. One cluster, Obekai was used as the intervention cluster for the implementation of the EHC through the FLTR strategy over a 3 year period [10]. The other, Kabula was used to implement the community driven and community owned health care service by encouraging the formation of income generating activities and community based organisations that were used to fund the level 1 and level 2 health care services over the 3 year period of implementations [12]. The remaining four clusters and affiliated dispensaries served as control clusters in which 6 monthly data on maternal and neonatal health indicators were collected over the 3 years of the study. In addition, baseline and end line/evaluation data was collected in these control clusters in years 1 and 5 respectively but no interventions were implemented.

### 4.2 Study clusters/locations

**Table:** 

	Name	County	Sub-County	Type	Sub county referral facility	County referral facility
1	Obekai	Busia	Teso South	Intervention Dispensary	Nambale subcounty hospital	Busia county hospital
2	Kabula	Bungoma	Bungoma South	Intervention Dispensary	Bungoma county hospital	Bungoma County Hospital
3	Nyaporo	Kakamega	Mumias	Control Dispensary	Makunga sub County hospital	Kakamega County referral Hospital
4	Kesses	Uasin Gishu	Eldoret South	Control Dispensary	Kesses sub County hospital	Uasin Gishu County referral Hospital
5	Matunda	Trans Nzoia	Trans Nzoia West	Control Dispensary	Matunda sub County hospital	Trans Nzoia County referral Hospital
6	Nyaru	Elgeyo Marakwet	Keiyo South	Control Dispensary	Chepkorio sub County hospital	Iten County referral Hospital

### 4.3 Inclusion criteria

• All pregnant women in the study clusters who gave written consent.
• All newborn babies, infants and under five children born during the life of the study within the respective study clusters.

### 4.4 Exclusion criteria

• Women not resident in the cluster during the study period.
• Newborns, infants and under-five children not resident in the study cluster.
• Infants and under-five children born before onset of the study in the study clusters
• Women living in the cluster that decline to give written consent

### 4.5 Data analysis

**Baseline and end line data** collection forms were developed and data collected and entered into a data base in the computer and analysed using the STATA version 12. Descriptive statistics were used to analyse and describe the quantitative data collected. An assessment of the maternal and child health indicators was then derived from this analysis including the status of the health system in the three arms of this study. Comparisons were made for various variables as outlined in the results section. The qualitative data collected from these three arms of the study has not been analysed.

**The *Intervention*** was designed and implemented as the EHC package using the FLTR strategy to improve access and quality of care of maternal and child health at tier 1 and 2 in one intervention cluster, Obekai in Busia County [10,11].

In the other cluster, Kabula in Bungoma, we worked with the community Health Units, village elders, Assistant chiefs and the chief in collaboration with the CHEWs and CHWs to establish income generating units (IGUs) and/or community based organizations (CBOs) in the cluster. The IGUs/CBO were set up by the communities themselves and run through governance structures established by the communities themselves. The funds from these IGUs/CBOs were used to incentivize the volunteer CHWs/CORPs to identify pregnant women and their newborns to the local dispensary. The primary care health facility committee worked with these CBOs and IGUs to assist in the funding of some activities including referral costs from the community to the primary care health facility and to the county referral facilities. These IGUs/CBOs were used to address the transport and birth plans requirements of the pregnant women, their newborns and infants born during the life of the project [10,12].

The four control clusters/dispensaries of Chesunet, Kiminini, Nyaru and Nyaporo did not have any intervention but continued to operate as normal within the existing health care delivery system as guided by the respective County Health systems. The study staff collected relevant MNCH data every 6 months from these control clusters and facilities. These four clusters served as the control arm and the data from the 4 was aggregated and the average was used for purposes of comparison with the other 2 intervention clusters.

## 5 Ethical considerations

The proposal was approved by the Institutional Research and Ethics Committee of Moi University and by the 6 County Health Management Teams (CHMTs). A unique identifier or study number was used to collect the data from each dyad of mother and infant and the name of the mother was not used. A separate record with names of the women in the study was kept confidentially by the lead investigator for reference as needed.

## 6 Consent process

The written consent was obtained from all women enrolled into the study who accepted to participate. Those mothers who were minors (age 12 years to age 17yrs) had their consents provided by their guardians or husbands. Illiterate eligible women had their thumb prints taken and witnessed by an independent adult. The IREC guidelines on confidentiality and on research among vulnerable groups were followed. For children under five, the mothers or guardians provided consent on their behalf.

## 7 Results

The comparison in various maternal and childhood indicators is between the two intervention clusters (Obekai, Kabula) and the Control clusters (Chesunet in Uasin Gishu County, Kiminini in Trans Nzoia County, Nyaru in Elgeyo Marakwet County and Nyaporo in Kakamega County) was done and the findings are shown in the below tables and figures. The four clusters results were put together as one by getting the aggregate result for each parameter since they acted as the control arm together.

There were a total of 6429 women enrolled in the study that participated and were followed up in the 6 clusters over the 3 year period of 2016 to 2018. Some of these women were still pregnant at the time of stopping the study in Sept 2018 and some had not become pregnant over the study period. There were 2787 newborns delivered by the women enrolled in the study over the 3 year study period who participated in the study in the 6 clusters.

There were 1128, 2676 and 2927 women included in this study in Obekai, Kabula and the control clusters respectively over the 3 year study period.

Since our objectives were outcome of pregnancy, facility access, utilization and quality of care, the demographic data of these women were not analyzed.

### 7.1 Maternal

#### 7.1.1 First ANC visit within the first 3 months

**Fig. 1:**
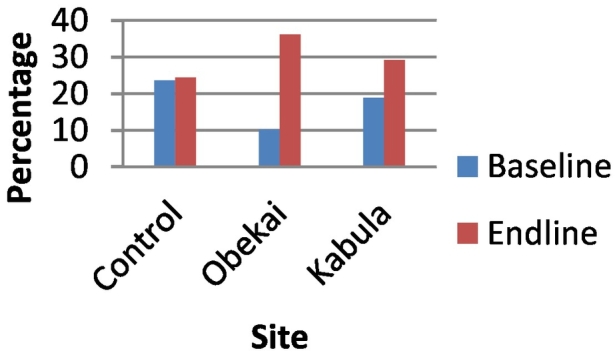
Timing of first ANC visit by site and time.

Fig. 1 shows the proportion of women who attended ANC in the first trimester by site and time. From the plot we observed that in the Control sites the proportion of women who attended ANC in the first trimester at baseline was 23.6% at baseline and increased to 24.4% at end line though the difference was not statistically significant (p-value = 0.427). In Obekai, the intervention site, the proportion was 10.2% at baseline increased to 36.2% at end line, the increase was statistically significant. In Kabula at baseline the proportion was 18.9% and 29.2% at end line and the increase was statistically significant (Table 1). We compared the difference in the differences between the Control sites, Obekai and Kabula and the results are shown in Table 2. We observed that the DID was statistically significant (95% CI: 0.201, 0.302) indicating a significant effect of the intervention on ANC attendance in the first trimester. (See Table 3, Table 4, Table 5.)

**Table 1:** Comparison of various maternal indicators across various sites between end line and baseline.

Arm	Baseline	Endoline	Difference	p-value	95% CI for the difference	
First ANC visit in the first 3 months						
Control	0.236	0.244	0.009	0.427	−0.012	0.029
Obekai	0.102	0.362	0.260	0	0.214	0.306
Kabula	0.189	0.292	0.103	0	0.069	0.137

Attendance of 4 plus ANC visits						
Control	0.458	0.628	0.169	0	0.146	0.193
Obekai	0.458	0.74	0.282	0	0.242	0.322
Kabula	0.612	0.67	0.058	0.003	0.020	0.096

Modern FP use						
Control	0.57	0.518	-0.051	0	−0.075	−0.028
Obekai	0.463	0.736	0.272	0	0.232	0.313
Kabula	0.653	0.815	0.162	0	0.128	0.196

Facility delivery						
Control	0.576	0.923	0.347	0	0.329	0.366
Obekai	0.515	0.887	0.372	0	0.337	0.407
Kabula	0.639	0.883	0.243	0	0.212	0.275

**Table 2:** Difference in differences for maternal indicators.

Comparison	DID	95% CI for DID	
First ANC visit in the first 3 months			
Obekaivs Control	0.251	0.201	0.302
Kabulavs Control	0.094	0.054	0.135
KabulavsObekai	−0.157	−0.215	−0.099

Attendance of 4plus ANC visits			
Obekaivs Control	0.113	0.067	0.159
Kabulavs Control	−0.111	−0.156	−0.067
KabulavsObekai	−0.224	−0.280	−0.169

Modern FP use			
Obekaivs Control	0.324	0.277	0.370
Kabulavs Control	0.213	0.172	0.255
KabulavsObekai	−0.110	−0.163	−0.058

Facility delivery			
Obekaivs Control	0.024	−0.015	0.064
Kabulavs Control	−0.104	−0.141	−0.067
KabulavsObekai	−0.128	−0.176	−0.081

**Table 3:** Comparison of intervention and control clusters for outcomes (*The four control clusters data was averaged for purposes of data analysis for comparison with the other 2 study arms).

Trends in vaccination coverage	2015	2016	2017	2018
BCG vaccination coverage				
Obekai	54%	*81%*	*89%*	*97%*
Kabula	61%	89%	97%	97%
Chesunet	28%	36%	15%	28%
Nyaporo	64%	61%	22%	27%
Nyaru	33%	36%	16%	17%
Kiminini	46%	29%	16%	35%

Polio 3 vaccination coverage				
Obekai	0%	*43%*	*87%*	*90%*
Kabula	0%	49%	62%	84%
Chesunet	51%	51%	25%	45%
Nyaporo	75%	85%	46%	71%
Nyaru	82%	82%	35%	52%
Kiminini	81%	74%	20%	52%

Penta 3 vaccination coverage				
Obekai	0%	*56%*	*70%*	*88%*
Kabula	0%	50%	62%	84%
Chesunet	51%	47%	26%	45%
Nyaporo	72%	86%	49%	73%
Nyaru	81%	80%	35%	53%
Kiminini	84%	77%	20%	51%

Fully immunized children at 9 months				
Obekai	6%	*41%*	*52%*	*85%*
Kabula	54%	54%	76%	76%
Chesunet	51%	52%	38%	63%
Nyaporo	65%	77%	39%	73%
Nyaru	59%	56%	42%	45%
Kiminini	54%	60%	29%	49%

Antenatal care attendance by pregnant women in the health facilities				
Obekai	46%	88%	88%	96%
Kabula	53%	69%	219%	330%
Chesunet	7%	7%	5%	8%
Nyaporo	45%	35%	21%	30%
Nyaru	66%	66%	26%	48%
Kiminini	16%	11%	5%	16%

ANC attendance 4+				
Obekai	45%	*48%*	*52%*	*59%*
Kabula	61%	27%	34%	55%
Chesunet	25%	34%	40%	42%
Nyaporo	26%	24%	13%	20%
Nyaru	14%	14%	12%	16%
Kiminini	11%	8%	6%	12%

Facility deliveries (Dispensary)				
Obekai	52%	*83%*	*86%*	*91%*
Kabula	64%	68%	79%	87%
Chesunet	57%	1%	1%	14%
Nyaporo	60%	59%	9%	27%
Nyaru	64%	0%	0%	0%
Kiminini	51%	3%	2%	2%

**Table 4:** Comparison of immunization indicators across sites between end line and baseline.

Arm	Baseline	Endoline	Difference	p-value	95% CI for the difference	
BCG						
Control	0.44	0.27	-0.17	0.00	-0.21	-0.14
Obekai	0.54	0.97	0.43	0.00	0.38	0.48
Kabula	0.61	0.97	0.36	0.00	0.33	0.39

Polio 3						
Control	0.75	0.55	-0.20	0.00	-0.24	-0.17
Obekai	0.43	0.9	0.47	0.00	0.41	0.53
Kabula	0.49	0.84	0.35	0.00	0.31	0.39

Pentavalent 3						
Control	0.76	0.56	-0.20	0.00	-0.24	-0.17
Obekai	0.56	0.88	0.32	0.00	0.26	0.38
Kabula	0.5	0.84	0.34	0.00	0.30	0.38

Measles						
Control	0.59	0.57	-0.02	0.40	-0.06	0.02
Obekai	0.41	0.85	0.44	0.00	0.37	0.52
Kabula	0.54	0.76	0.22	0.00	0.18	0.26

**Table 5:** Difference in Differences for the immunization indicators.

Comparison	DID	95% CI for DID	
BCG			
Obekaivs Control	0.60	0.54	0.67
Kabulavs Control	0.53	0.48	0.58
KabulavsObekai	-0.07	-0.13	-0.01

Polio 3			
Obekaivs Control	0.67	0.60	0.74
Kabulavs Control	0.55	0.50	0.61
KabulavsObekai	-0.12	-0.19	-0.05

Pentavalent 3			
Obekaivs Control	0.52	0.46	0.59
Kabulavs Control	0.54	0.49	0.60
KabulavsObekai	0.02	-0.05	0.09

Measles			
Obekaivs Control	0.46	0.38	0.54
Kabulavs Control	0.24	0.18	0.30
KabulavsObekai	-0.22	-0.31	-0.14

#### 7.1.2 Attendance of 4 or more ANC visit

**Fig. 2:**
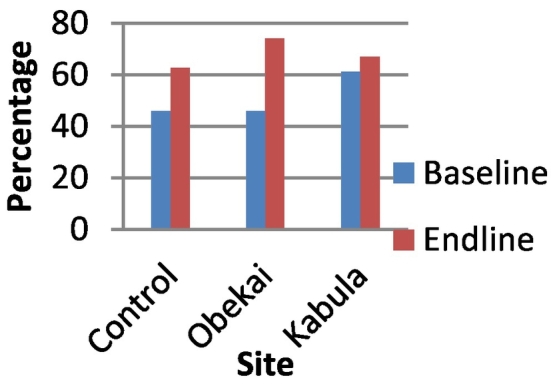
Percentage of women attending 4 plus ANC visits by site and time.

In the control sites the proportion of women who reported to have attended 4 or more ANC visits in the last pregnancy prior to the survey was 45.8% at baseline and 62.8% at end line (Fig. 2). In Obekai it was 45.8% at baseline and 74% at end line while in Kabula it was 61.2% at baseline and 67% at end line. In all the sites the increase was statistically significant, p-value <0.05 (Table 1). When we compared the differences in difference we observed that there was a significant improvement in ANC attendance in Obekai compared to the controls (95%CI: 0.067,0.159) as shown in Table 1 and Fig. 2.

#### 7.1.3 Modern contraceptive use

**Fig. 3:**
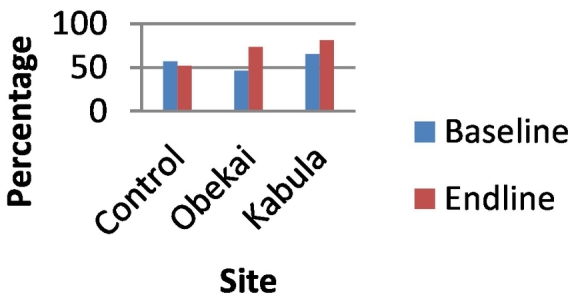
Percentage of women using modern contraceptive by site and time.

In the baseline survey the percentage of women who reported modern contraceptive use was 57% in the control site, 46.35 in Obekai and 65.3% in Kabula. At end line there was a significant drop in the control clusters to 51.8% (p-value<0.05) while in Obekai and Kabula there was an increase to 73.6% and 81.5% respectively (p-value<0.05) as shown in Table 1. In terms of treatment effect using the DID there was a significant improvement in uptake of modern contraceptive when we compared Obekai and the control site (95%CI:0.277,0.370) as shown in Table 1 and Fig. 3.

#### 7.1.4 Facility delivery

**Fig. 4:**
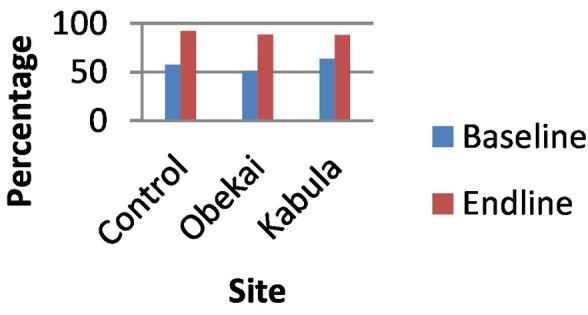
Percentage of women delivering in a health facility by site and time.

**Fig. 5:**
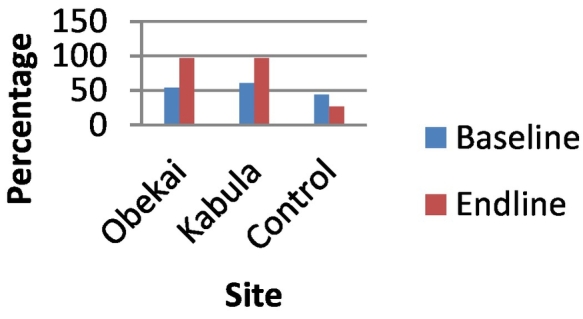
BCG vaccination coverage comparisons at baseline and end line.

**Fig. 6:**
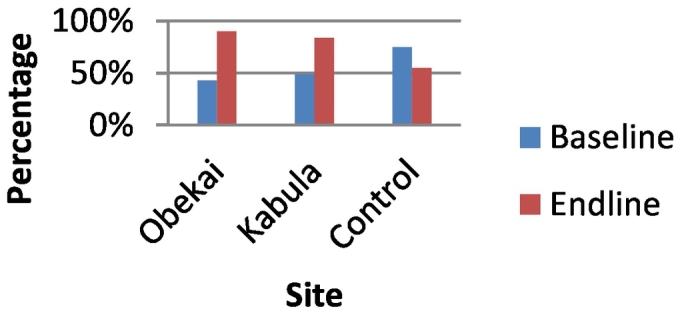
Polio 3 vaccination coverage comparisons at baseline and endline.

**Fig. 7:**
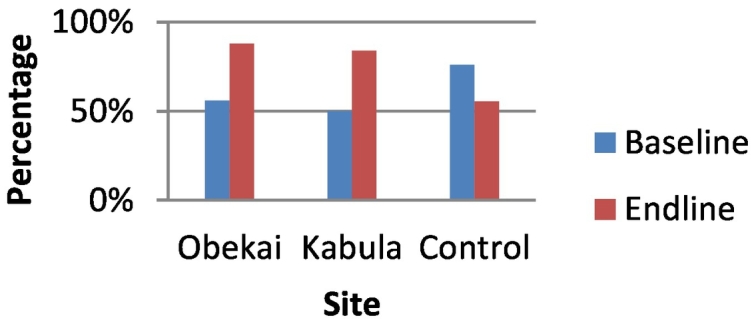
Pentavalent 3 vaccination coverage comparisons at baseline and end line.

**Fig. 8:**
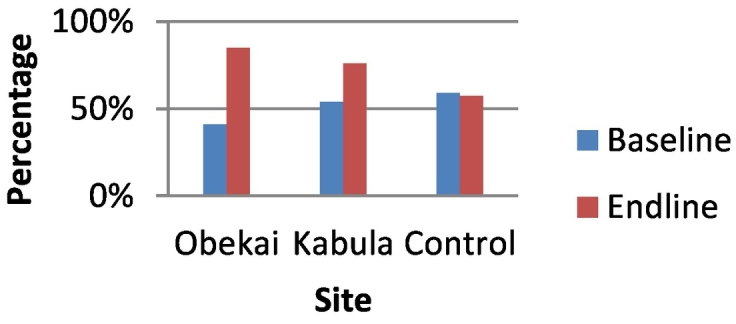
Measles vaccination coverage comparisons at baseline and end line.

In the control site the proportion who reported hospital delivery in their last birth was 57.6% at baseline and there was a statistically significant increase to 92.3% at end line. In Obekai there was a statistically significant increase from 51.5% at baseline to 88.7% at end line while in Kabula there was an increase from 63.9% to 88.3% which was statistically significant. In terms of DID there was no statistically significant difference in the improvement in facility deliveries when we compared Obekai and the control site 95%CI:-0.015,0.370. This is shown in Table 1 and Fig. 4. (See Fig. 5, Fig. 6, Fig. 7, Fig. 8.)

### 7.2 Neonatal indicators

#### 7.2.1 BCG vaccination rates

The BCG vaccination increased in Obekai and Kabula over the study period but declined in the 4 control locations. There was a 21% and a 19% increase in immunization coverage in Obekai and Kabula respectively with a reduced coverage of 12% in the control clusters.

#### 7.2.2 Polio 3 immunization coverage

The Polio 3 vaccination increased in Obekai and Kabula over the study period but declined in the 4 control locations. There was a 24% and a 17% increase in immunization coverage in Obekai and Kabula respectively with a reduced coverage of 10% in the control clusters.

#### 7.2.3 Pentavalent 3 vaccination coverage

The Pentavalent 3 vaccination increased in Obekai and Kabula over the study period but declined in the 4 control locations. There was a 31% and a 32% increase in immunization coverage in Obekai and Kabula respectively with a reduced coverage of 20% in the control clusters.

#### 7.2.4 Measles vaccination coverage

The Measles vaccination increased in Obekai and Kabula over the study period but declined in the 4 control locations. There was a 45% and a 22% increase in immunization coverage in Obekai and Kabula respectively with a reduced coverage of 2% in the control clusters.

## 8 Discussion

This study was conducted as an innovation within the context of the health systems six pillars as described by WHO with the objective of linking communities and the primary care facilities. The overall goal was to improve access and utilization of the health care system by communities and to enhance the referral system in the health care delivery system [8,9,11].

The findings of this study on maternal indicators are similar to those found in studies that used the health systems approach in maternal health care, Savings Mothers Giving Life (SMGL) project in Uganda and Zambia [[13], [14], [15]]. Similar findings were found by Olayo [16] in Kenya in 2014 on the community strategy implementation in Kenya but not on the health systems as outlined by WHO.

In this study the WHO Health System pillars were applied as contextualized in our protocol and in our earlier publications [[10], [11], [12]].

The WHO pillars of the health systems include governance of the health system in which there should an organized management structure in all cadres of health workers with a leader who may be a doctor, a Senior Nurse or a clinical Officer. Currently dispensaries are headed by a nurse but this does not allow for all clinical services to be offered as required by a holistic health system. A dispensary needs to be headed by a doctor or a Clinical Officer in order to improve clinical care thus reducing referrals to higher health facilities which are distant to the communities.

The other pillar was the continuous and consistent supply of commodities, drugs and dressings needed for the provision of quality health care. The erratic supply of these hampers access and utilization of health services in this level of health care. Our Obekai site implemented this throughout the three years with good results in access, utilization and provision of quality health with satisfactory feedback by the clients that attended the facility.

The importance of the Health management Information Systems (HMIS) as one of the pillars cannot be overemphasized as it makes instant retrieval of client data at follow up. In Obekai we introduced the HMIS which made our follow up at the facility extremely smooth and it was easy to trace and track drop out and get them to the facility for care.

The human resource pillar is very important in the health system. In Kenya we have 13/100,000 ratio of human resource to population while the WHO recommends a 23/100,000 ratio. This shortage in health care human resources creates reduced access, reduced utilization and inadequate provision of quality health services. In Obekai we added a Clinical Officer and two nurses to the dispensary to address this pillar and the shortage.

Financing of the health systems is a very significant pillar since most commodities need to be purchased in the facilities. In Obekai dispensary we had a budget to cater for all the needs of the facility and it did not encounter any stock outs of medical commodities needed including power supply and water supply. This improved the access and utilization of the facility over the three years.

Finally, the service delivery pillar of the health system needs to be implemented in a consistent manner and as per the provisions of the standard operating procedures (SOPs) and guidelines of the Ministry of Health and the WHO. We ensured these guidelines, SOPS and procedures were implemented in Obekai dispensary and this also contributed the improvement of the quality of health care which led to improved access and utilization of the health services at the facility.

The study also looked at the principles of the three delays in health care outlined by Thaddeus in his studies [6]. We applied these principles by Thaddeus together with the WHO pillars of the health system during the implementation of the study [7,8,17]. We ensured that transport was available to refer mothers and infants from their homes to the facility and for referrals to the higher level facilities. We also ensured the health services were always provided and the quality of the services was as prescribed by the Ministry of Health as per its SOPs and guidelines. We also addressed the concept health seeking behavior through the CHWs who ensured the community was made aware of the signs and symptoms of common illnesses and the need to seek health care in the facility when they felt unwell.

From the results shown above we observe that in the Control sites the proportion of women who attended ANC in the first trimester at baseline increased at the end of the study though the difference was not statistically significant. In Obekai the main intervention site where the research team participated actively in the implementation of the study as per protocol, the increase in ANC attendance at 3 months pregnancy was high and statistically significant. In Kabula, where the implementation was community driven and owned, the increase was also significant though lower than the Obekai location. When the difference in the differences (DID) between the Control sites and Obekai and Kabula, we observed that the DID was statistically significant indicating a significant effect of the intervention on ANC attendance in the first trimester in both Obekai and Kabula communities.

In the control sites the proportion of women who reported to have attended 4 or more ANC visits in the last pregnancy prior to the survey increased slightly but the increase was more significant in the Obekai and Kabula locations. When the differences using the DID was done there was a significant improvement in ANC attendance in Obekai compared to the Kabula and the controls.

In the baseline survey the percentage of women who reported modern contraceptive use was markedly decreased in the control locations than in Obekai and Kabula. In terms of treatment effect using the DID there was a significant improvement in uptake of modern contraceptive in Obekai and Kabula when compared the control sites where there was a decline in contraceptive use.

There was an increase in the proportion of women who reported hospital delivery in their last birth in control locations which was a little lower than in Kabula. However, these levels in control locations and in Kabula were lower than in Obekai. However, when the DID analysis was done there was no statistically significant difference in the improvement in facility deliveries in all the locations (Obekai, Kabula and the controls).

When vaccinations coverage was analyzed the BCG vaccination increased in Obekai and Kabula over the study period but declined in the 4 control locations. The Polio 3 vaccination increased in Obekai and Kabula over the study period but declined in the 4 control locations. The Pentavalent 3 vaccination increased in Obekai and Kabula over the study period but declined in the 4 control locations. The Measles vaccination increased in Obekai and Kabula over the study period but declined in the 4 control locations.

These observations suggest that the health systems when applied conventionally or through a community oriented approach, have a positive impact on quality, access and utilization of health services. These observations are similar to other related studies [13,15], though these other studies were not comparative nor quasi-experimental in design. It is therefore proposed that where there are intentions to implement Universal Health Coverage programs or a health system in developed and developing countries the application of the health systems approach as outlined in this paper be considered. The health systems approach should also be considered in policy formulations in countries that review their health services delivery programs in the future or intend to reform their health services delivery models.

From the findings in this study, it is clear that the holistic application of the WHO model of the health systems which has the components of leadership and governance, availability of medical commodities, adequacy of human resources for health, HMIS, adequate financing and provision quality health care service delivery has a significant impact on access, utilization and quality health care provision in facilities and the community. This was applied holistically in the Obekai cluster, partially in the Kabula cluster and not at all in the control clusters and the findings in the Obekai cluster suggest and support the need to apply this health systems package in the provision of health care especially in the primary care facilities.

There are few experimental or quasi-experimental studies that have been conducted in the field of health systems. We were not able to find comparative studies that have been done locally and globally. The findings in this study would therefore lay the foundation upon which larger and more elaborate studies of this caliber should be designed and conducted. In his analysis of quasi-experiments in public health, Bärnighausen [18] confirms that strong causal analysis of health policy impacts is key to good decision-making, and ultimately good population health. He further states that randomized controlled experiments in the health sector are commonly not feasible because of ethical, political or financial constraints. Quasi-experiments provide a good opportunity for causal analyses in these situations, and they have the added advantage that they avoid the artificiality that trials introduce into the study context because of the trial-associated intervention and measurement.

### 8.1 Limitations of the study

The major limitation in this study was missing and inconsistent data in the control clusters since we did not do any intervention in these clusters but only collected data on quarterly basis. Due to missing data in the control clusters some of the maternal and childhood health indicators could not be measured and compared and therefore these are not presented in this publication. These include rates of pregnancy outcomes (birth asphyxia, neonatal sepsis, neonatal mortality, maternal mortality, near miss maternal morbidity among pregnant women) and exclusive breastfeeding rate, Infant and Young Children Feeding (IYCF) and IMCI parameters and anthropometric indicators.

## 9 Conclusions

Use of the health systems approach in health care provision provides a holistic improvement in access and utilization of health services and improvement in health indicators.

## Recommendations

The Health systems approach in health care provision as envisaged by the WHO should be applied in the implementation of health care delivery programs including universal health coverage locally, regionally and globally.

More studies should be carried out on the health system implementation especially on larger scales.

## Ethics approval and consent to participate

The proposal was approved by the Research and Ethics committee of Moi University.

## Consent for publication

The authors have all approved the publication of the proposal.

## Availability of data and material

Not applicable.

## Funding

Funding was obtained from NACOSTI under the IDRC funding mechanism for Research Chairs in Kenya.

## Authors' contributions

All the authors participated in the design and development of the proposal. FOE conceived the idea, provided the pediatrics facts and wrote the draft proposal, EOW provided the guidance on the obstetric facts of the proposal, AM provided the statistical details and support, DA provided the behavioral aspects of the proposal, JT provided the community and referral strategic aspects of the proposal and MN deputized the corresponding author and provided the demographic and health systems aspects of the proposal.

## Declaration of Competing Interest

“This work was carried out with the financial support from the 10.13039/501100005864National Commission for Science, Technology and Innovation (NACOSTI) and the 10.13039/501100000193International Development Research Centre (IDRC) Canada. The views expressed in this work are those of the creators and do not necessarily represent those of the 10.13039/501100005864National Commission for Science, Technology and Innovation, and the 10.13039/501100000193International Development Research Centre, Canada or their Board of Governors.”
